# Neurogenesis, Neurodegeneration, Interneuron Vulnerability, and Amyloid-β in the Olfactory Bulb of APP/PS1 Mouse Model of Alzheimer's Disease

**DOI:** 10.3389/fnins.2016.00227

**Published:** 2016-05-30

**Authors:** Carlos De la Rosa-Prieto, Daniel Saiz-Sanchez, Isabel Ubeda-Banon, Alicia Flores-Cuadrado, Alino Martinez-Marcos

**Affiliations:** Neuroplasticity and Neurodegeneration Laboratory, CRIB, Ciudad Real Medical School, Universidad de Castilla-La ManchaCiudad Real, Spain

**Keywords:** adult neurogenesis, calbindin, calretinin, parvalbumin, somatostatin

## Abstract

Alzheimer's disease (AD) is the most prevalent neurodegenerative disease, mostly idiopathic and with palliative treatment. Neuropathologically, it is characterized by intracellular neurofibrillary tangles of tau protein and extracellular plaques of amyloid β peptides. The relationship between AD and neurogenesis is unknown, but two facts are particularly relevant. First, early aggregation sites of both proteinopathies include the hippocampal formation and the olfactory bulb (OB), which have been correlated to memory and olfactory deficits, respectively. These areas are well-recognized integration zones of newly-born neurons in the adult brain. Second, molecules, such as amyloid precursor protein (APP) and presenilin-1 are common to both AD etiology and neurogenic development. Adult neurogenesis in AD models has been studied in the hippocampus, but only occasionally addressed in the OB and results are contradictory. To gain insight on the relationship between adult neurogenesis and AD, this work analyzes neurogenesis, neurodegeneration, interneuron vulnerability, and amyloid-β involvement in the OB of an AD model. Control and double-transgenic mice carrying the APP and the presenilin-1 genes, which give rise amyloid β plaques have been used. BrdU-treated animals have been studied at 16, 30, 43, and 56 weeks of age. New-born cell survival (BrdU), neuronal loss (using neuronal markers NeuN and PGP9.5), differential interneuron (calbindin-, parvalbumin-, calretinin- and somatostatin-expressing populations) vulnerability, and involvement by amyloid β have been analyzed. Neurogenesis increases with aging in the granule cell layer of control animals from 16 to 43 weeks. No neuronal loss has been observed after quantifying NeuN or PGP9.5. Regarding interneuron population vulnerability: calbindin-expressing neurons remains unchanged; parvalbumin-expressing neurons trend to increase with aging in transgenic animals; calretinin-expressing neurons increase with aging in transgenic mice and decrease in control animals and neurogenesis is higher in control as compared to transgenic animals at given ages, finally; somatostatin-expressing neurons of transgenic mice decrease with aging and as compared to controls. Amyloid β aggregates with aging in the granule cell layer, which may be related to the particular involvement of somatostatin-expressing cells.

## Introduction

Alzheimer's disease (AD) is the most prevalent neurodegenerative disease and the main cause of dementia (Reiman, 2014). It is characterized by cognitive deficits and nowadays treatments are palliative. Two associated proteinopathies yield aggregates into the brain: tau protein that form intracellular neurofibrillary tangles and insoluble forms of amyloid β (Aβ) peptides that assembles into extracellular plaques (Goedert and Spillantini, 2006; Ballard et al., 2011). Neuropathological staging of these deposits show that early sites of involvement include the OB and the hippocampal formation (Ohm and Braak, 1987; Braak and Braak, 1991; Braak et al., 2006; Attems et al., 2014; Braak and Del Trecidi, 2015). These areas correlate with initial symptoms, namely olfactory (Devanand et al., 2015) and declarative memory deficits (Jahn, 2013).

Interestingly, the subventricular zone of the lateral ventricles (SVZ) and the hippocampal subgranular zone (SGZ) were described as the two main neurogenic niches giving rise to newly-born neurons migrating and integrating into the adult olfactory bulb (OB) and the dentate gyrus (DG) of the hippocampus, respectively (Altman, 1962; Altman and Das, 1965; Luskin, 1993; Lois and Alvarez-Buylla, 1994). Decades later, the functional significance of new neurons integrated into adult OB (Lepousez et al., 2013) and DG (Deng et al., 2010) is only partially known.

AD and adult neurogenesis are not only linked by common sites where early pathology occurs and newly-born neurons integrate in the preexisting circuitry, but share a number of common molecules to both processes (Kaneko and Sawamoto, 2009; Lazarov and Marr, 2010, 2013; Lazarov et al., 2010; Mu and Gage, 2011; Winner et al., 2011). Increasing evidence suggest that molecular players in Alzheimer disease, including amyloid precursor protein (APP) and presenilin 1 (PS1) and its metabolites, play a role in adult neurogenesis (Lazarov and Marr, 2010). Soluble APPα regulate neural progenitor cell proliferation. Thus, miscleavage of APP would readily influence on developmental and postnatal neurogenesis, which could contribute to cognitive deficits characterizing AD (Lazarov and Demars, 2012). On the other hand, PS1 is not only prominently expressed in the embryonic brain but is also a crucial regulator of Notch and Wnt signaling (Winner et al., 2011), key pathways in neural differentiation. In addition, polymorphisms in the apolipoprotein E (apoE, the ε4 isoform) gene show the most significant effects on relative genetic risk of AD. Experiments in transgenic mice carrying human apoE4 have shown apoptosis of neural progenitor cells after environmental enrichment suggesting that apoE4 is somewhat compromising neurogenesis (Levi and Michaelson, 2007).

Data on neurogenic rate changes in AD models are quite variable depending on the different transgenic mice, experimental conditions, and markers analyzed; and, it is not always obvious to distinguish it from changes due to physiological aging (Lazarov and Marr, 2010; Winner et al., 2011). Data in the model used in the present study (APPswe/PS1ΔE9) show reduced hippocampal neurogenesis (Verret et al., 2007; Niidome et al., 2008). This impairment in neurogenesis take place early in life long before amyloid deposition suggesting that the decrease of neurogenic rate may be a contributor factor rather than a result of neural dysfunction (Demars et al., 2010). Interestingly, the reduction in neuroblasts, as confirmed by quantitative Western blot analysis of doublecortin content, was restricted to the hippocampal but not to the OB neurogenic system (Zhang et al., 2007).

Data from different authors and our previous results indicate a differential interneuron vulnerability in postmortem tissue from Alzheimer's patients and in transgenic mice model (Fonseca and Soriano, 1995; Solodkin et al., 1996; Brady and Mufson, 1997; Leuba et al., 1998; Iritani et al., 2001; Saiz-Sanchez et al., 2010, 2012, 2013, 2015, 2016).

Therefore, the present study aims at characterizing the neurogenic process in the OB of APP/PS1 mice by analyzing the neurogenic and neurodegeneration rates and the role Aβ in the survival of new and preexisting interneuron populations.

## Materials and methods

### Experimental animals

For this study, 20 female hemizygous double transgenic mice (B6C3-Tg-APPswe, PSEN1dD9-85Dbo/J) model of AD and 20 female non-carrier mice have been used (004462, The Jackson Laboratory, USA). These transgenic mice express a chimeric mouse/human APP (Mo/HuAPP695swe) and a mutant human presenilin 1 (PS1-dD9), each controlled by independent mouse prion protein (PrP) promoter elements (Jankowsky et al., 2001, 2004). The APPswe/PSEN1dD9 (APPxPS1) mouse model is characterized by increasing Aβ levels with aging (Jankowsky et al., 2004; Van Groen et al., 2006). Four experimental groups of five transgenic and five non-carrier animals were stablished according to survival times (16, 30, 43, and 56 weeks). The animals were housed on a standard 12/12 h light/dark cycle, at 21°C with food and water *ad libitum*. All of the animal research procedures described herein were in agreement with European (Directive 2010/63/EU) and Spanish (RD 53/2013) legislation on the protection of animals used for scientific purposes. All experiments described were approved by the Ethical Committee of Animal Research of the University of Castilla-La Mancha (grant BFU2010-15729).

### Bromodeoxyuridine administration, perfusion, and sectioning

BrdU (5-bromo-2′-deoxyuridine, Fluka, Madrid, Spain) administration included four i.p. doses (at 2-h intervals) of 10 mg/mL BrdU in phosphate-buffered saline (PBS, 0.15 M NaCl, 0.01 M sodium phosphate pH 7.4) totalizing a dose of 200 mg/kg in 1 day. This dose was employed following previous results in our laboratory to optimize labeling without increasing apoptosis (Martínez-Marcos et al., 2000a,b; Martinez-Marcos et al., 2005; De La Rosa-Prieto et al., 2009). Two week afterwards, animals were anesthetized with a combined dose of ketamine hydrochloride (Ketolar, Parke-Davis, Madrid, Spain, 1.5 mL/kg, 75 mg/kg), and xylazine (Xilagesic, Calier, Barcelona, Spain, 0.5 mL/kg, 10 mg/kg) and perfused with saline solution followed by 4% w/v paraformaldehyde fixative in phosphate buffer (0.1 M sodium phosphate pH 7.2). Brains were postfixed in 4% w/v paraformaldehyde, cryoprotected in 30% w/v sucrose, and the olfactory bulbs frontally sectioned (50 μm) using a freezing sliding microtome (Microm HM450). Sections were consecutively collected into 96-well-plates and maintained at 4°C in preserving solution (PBS containing 20% v/v glycerol and 30% v/v ethylene glycol) for further processing.

### Immunofluorescence procedures

Six sections from rostral to caudal OB of each animal were chosen (separated 400 um) and rinsed overnight with Tris-buffered saline (TBS; 0.15 M NaCl, 0.05 M Tris, HCl pH 7.6) and blocked with 10% v/v normal donkey serum (NDS; Vector Laboratories, Burlingame, CA) in TBS for 60 min at room temperature. Sections were then incubated overnight with rabbit anti-amyloid beta (Aβ, 1:250, Cell Signaling, 2454, MA, USA), goat anti-calretinin (CR, 1:1000, Santa Cruz, sc-11644, CA, USA), monoclonal mouse anti-calbindin D-28k (CB, 1:5000, Swant, 300, Switzerland), goat anti-somatostatin (SST, 1:1000, Santa Cruz, sc-7819, CA, USA), goat anti-parvalbumin (PV,1:2000, Swant, PVG-213, Switzerland), mouse monoclonal PGP9.5 (PGP9.5 13C4/I3C4, 1:1000, abcam, ab8189, Cambridge, MA, USA), or rabbit anti-NeuN (NeuN, 1:500, abcam, ab104225, Cambridge, MA, USA) diluted in TBS with 5% v/v normal goat serum and 0.3% Triton X-100 at 4°C. Then, sections were incubated for 2 h at room temperature with Alexas 488, 555, 568, or 647 anti-multiple species (1:200 in TBS with 2% of normal goat serum and 0.2% Triton X-100; Invitrogen, Eugene, OR).

Sections were then rinsed with TBS and incubated in ice-cold paraformaldehyde 4% v/v for 15 min. Rinsed again and incubated in 2N HCL at 37° during 1 h. After rinsed several times, sections were incubated overnight with mouse anti-BrdU (BrdU,1:40, Dako, M0744, Glostrup, Denmark). Sections were subsequently incubated for 2 h at room temperature with Alexa 488, anti-mouse (1:200 in TBS with 2% of normal goat serum and 0.2% Triton X-100; Invitrogen, Eugene, OR), and counterstained using DAPI (1 μg/ml in TBS, Santa Cruz, SC-3598) for 5 min in the dark.

### Analysis of labeled cells

The images of different fluorophores were analyzed using ImageJ and ZEN software from Zeiss using the profile and ortho tools of ZEN software. GraphPad Prism® v.6 (San Diego, CA, USA) was used for statistical analyses. Kolmogorov–Smirnov and Wald–Wolfowitz tests were carried out to analyze the normality and randomness of the sample (*P* > 0.05). Statistical comparisons were performed using an unpaired two-tailed *t*-test, or one-way or two-way ANOVA followed by Bonferroni and Tukey *post-hoc* tests to estimate the significance of differences between age groups, markers, areas, WT, and TG animals. All data are represented as mean ± SEM. Differences were regarded as statistically significant at ^*^ or #*P* < 0.05, ^**^ or ##*P* < 0.01, ^***^ or ###*P* < 0.001, and ^****^P < 0.0001.

## Results

**Figure 1:**
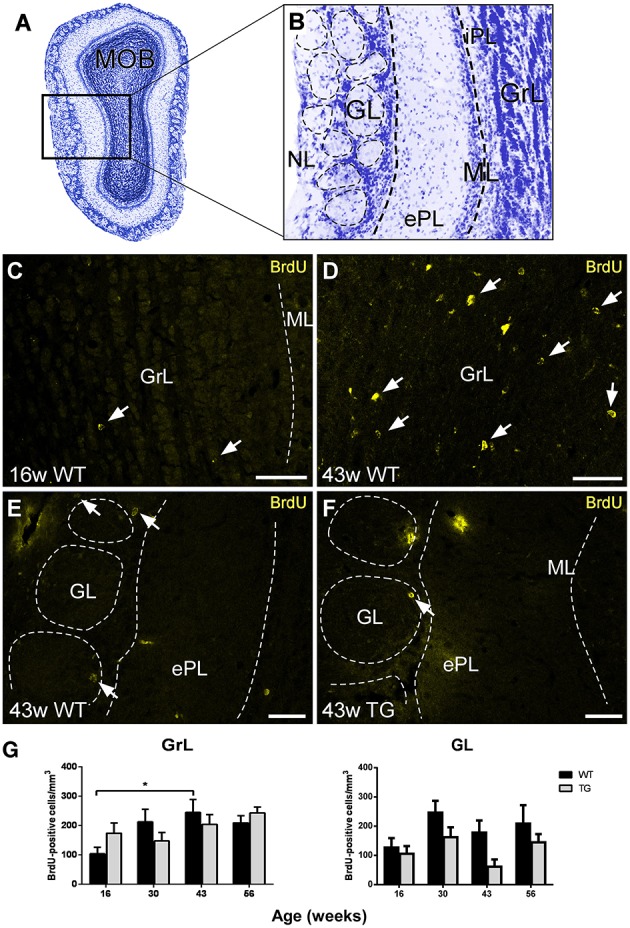
**Analysis of neurogenesis in the olfactory bulb by quantification of bromodeoxyuridine-labeled cells. (A,B)**, Nissl staining of a mouse main olfactory bulb showing all cell layers where analysis has been carried out. **(C–F)**, examples of bromodeoxyuridine-positive cells at different ages, in different layers, and in wild type and transgenic animals illustrating statistical analysis. **(G)**, graphics illustrating the estimation of bromodeoxyuridine-positive cells/mm^3^ in control and transgenic animals in different layers with aging. Calibration bar, 50 μm. White arrows point to labeled cells. ^*^P < 0.05.

The aim of the present report has been to study in depth the relationship between adult neurogenesis and AD by analyzing neurogenic rate, neurodegeneration, interneuron vulnerability, and Aβ involvement in the OB of control and transgenic mice over time. The different layers of the main OB (MOB; Figure 1A) have been grouped for analysis: granule (GrL) and internal plexiform (iPL), mitral (ML), and external plexiform (ePL), and glomerular (GL), and nerve (NL) layers (Figure 1B).

### Analysis of BrdU-labeled cells

The labeling of BrdU-positive cells was mainly concentrated in the GrL and GL of control and transgenic animals at different ages (Figures 1C–F). The analysis of BrdU-labeled cells over time revealed an increasing trend in the GrL with aging that was statistically significant between 16 (Figure 1C) and 43 (Figure 1D) weeks in control animals (Figure 1G) [Two-way ANOVA genotype vs. age: Interaction *F*_(3, 62)_ = 1.846; *p* = 0.1482; Age *F*_(3, 62)_ = 3.220; *p* = 0.0287; Genotype *F*_(1, 62)_ = 0.0002612; *p* = 0.9872]. This trend was not observed in the GL (Figure 1G) [Two-way ANOVA genotype vs. age: Interaction *F*_(3, 63)_ = 0.5647; *p* = 0.6403; Age *F*_(3, 63)_ = 2.676; *p* = 0.0547; Genotype *F*_(1, 63)_ = 7.260; *p* = 0.0090].

Regarding genotype comparison, no clear differences were observed in the GrL, but in the GL, the number of BrdU-labeled cells was in general lower in transgenic (Figure 1F) as compared to control (Figure 1E) animals.

### Analysis of NeuN- and PGP9.5-labeled cells

**Figure 2:**
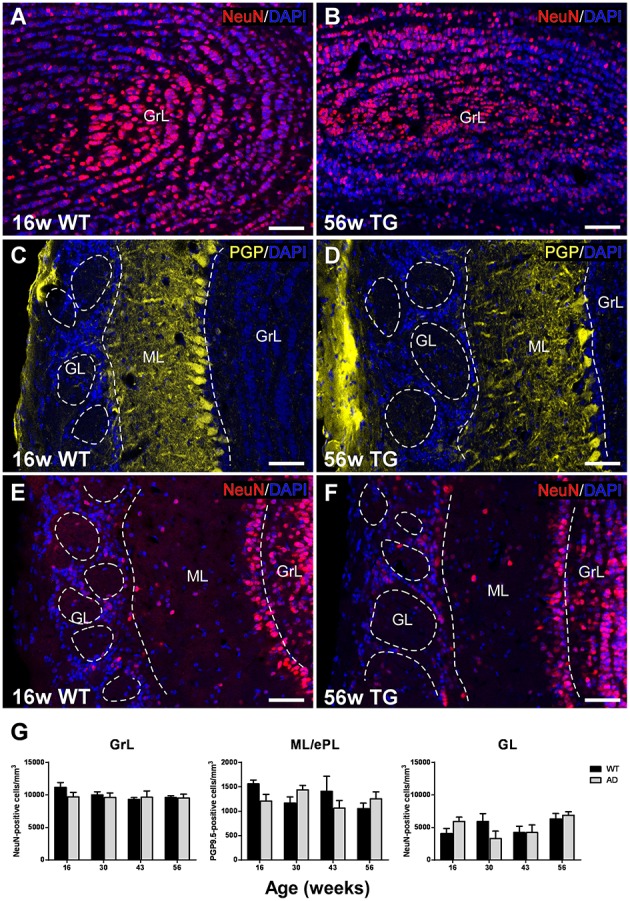
**Analysis of neurodegeneration in the olfactory bulb by quantification of NeuN- and PGP9.5-labeled cells**. Confocal images showing NeuN-positive **(A,B,E,F)** and PGP9.5-positive cells **(C,D)** counterstained with DAPI. **(G)**, graphics illustrating the estimation of NeuN- and PGP9.5-positive cells/mm^3^ in control and transgenic animals in the different layers with aging. Calibration bar, 50 μm.

In order to evaluate a possible neuronal loss, two neural markers have been used in the OB of control and transgenic mice: NeuN has been used to label neurons in GrL (Figures 2A,B) and GL (Figures 2E,F), and PGP9.5 in ML (Figures 2C,D) since not all neural populations are labeled by commercial neural markers (see Supplementary Figure 1) (Bagley et al., 2007; Bianchi et al., 2014).

Statistical analysis showed no significant changes over time and genotype or between control and transgenic animals in the GrL (Figure 2G) [Two-way ANOVA (genotype vs. age): Interaction *F*_(3, 28)_ = 0.7079; *p* = 0.5554; Age *F*_(3, 28)_ = 0.9086; *p* = 0.4494; Genotype *F*_(1, 28)_ = 0.7859; *p* = 0.3829], ML [Two-way ANOVA (genotype vs. age): Interaction *F*_(3, 28)_ = 2.443; *p* = 0.0849; Age *F*_(3, 28)_ = 0.8184; *p* = 0.4946; Genotype *F*_(1, 28)_ = 0.2668; *p* = 0.6095] or GL [Two-way ANOVA (genotype vs. age): Interaction *F*_(3, 28)_ = 2.054; *p* = 0.1291; Age *F*_(3, 28)_ = 2.496; *p* = 0.0803; Genotype *F*_(1, 28)_ = 0.005049; *p* = 0.9439].

### Analysis of interneuron markers

**Figure 3:**
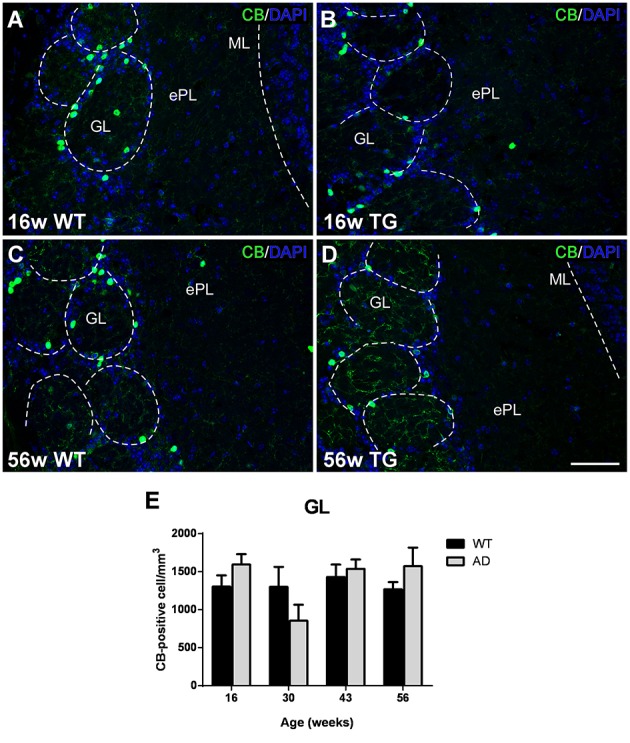
**Vulnerability analysis of calbindin-expressing cells in the olfactory bulb. (A–D)**, Confocal images showing calbindin-positive cells in the glomerular layer of control and transgenic animals at different ages. **(E)**, graphics illustrating the estimation of calbindin-positive cells/mm^3^ in control and transgenic animals in the glomerular layer with aging. Calibration bar, 50 μm.

Calbindin-positive neurons were mostly concentrated in the GL in both control (Figures 3A,C) and transgenic (Figures 3B,D) animals. No significant changes were observed with aging (Figures 3A,B vs. Figures 3C,D) or genotype (Figures 3A,C vs. Figures 3B,D) as demonstrated statistically (Figure 3E) [Two-way ANOVA (genotype vs. age): Interaction *F*_(3, 28)_ = 1.679; *p* = 0.1940; Age *F*_(3, 28)_ = 1.942; *p* = 0.1457; Genotype *F*_(1, 28)_ = 0.2197; *p* = 0.6429].

Most parvalbumin-labeled neurons appeared in the ML/ePL in control (Figures 4A,C) and transgenic mice (Figures 4B,D). The expression appear to increase from 16 (Figures 4A,B) to 56 (Figures 4C,D) in both control and transgenic animals. Statistical analysis demonstrated a significant increase from 16 to 43 and 56 weeks in transgenic animals (Figure 4E). No changes were significant between control and transgenic animals [Two-way ANOVA (genotype vs. age): Interaction *F*_(3, 28)_ = 2.026; *p* = 0.1331; Age *F*_(3, 28)_ = 3.617; *p* = 0.0252; Genotype *F*_(1, 28)_ = 0.6551; *p* = 0.4251].

**Figure 4:**
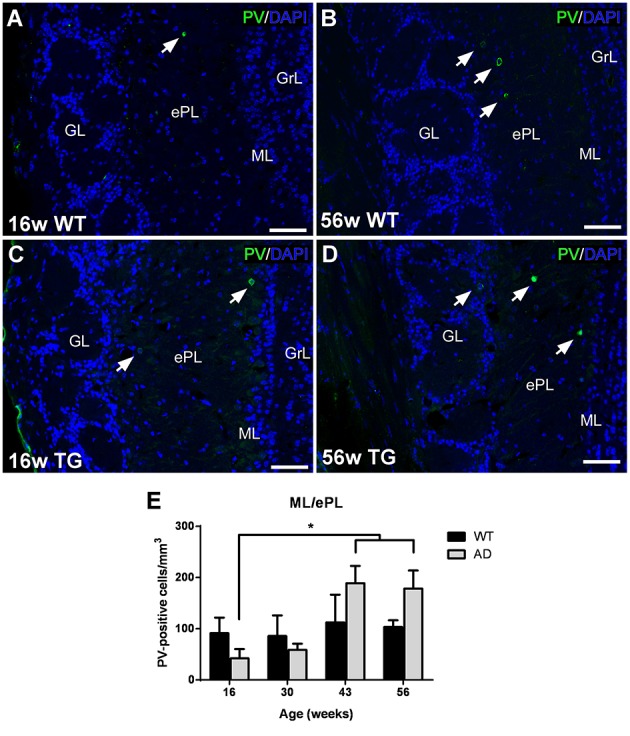
**Vulnerability analysis of parvalbumin-expressing cells in the olfactory bulb. (A–D)**, confocal images showing parvalbumin-positive cells in the mitral/external plexiform layer of control and transgenic animals at different ages. **(E)**, Graphics illustrating the estimation of parvalbumin-positive cells/mm^3^ in control and transgenic animals in the mitral/external plexiform layer with aging. Calibration bar, 50 μm. White arrows point to labeled cells. ^*^P < 0.05.

**Figure 5:**
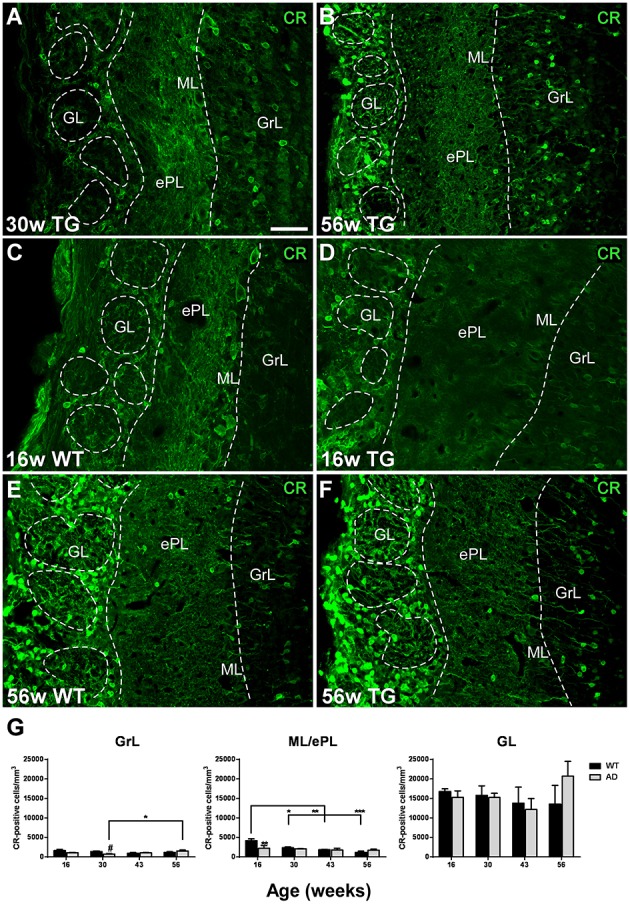
**Vulnerability analysis of calretinin-expressing cells in the olfactory bulb. (A–F)**, confocal images showing calretinin-positive cells in the different layers of control and transgenic animals at distinct ages. **(G)**, graphics illustrating the estimation of calretinin-positive cells/mm^3^ in control and transgenic animals in the different layers with aging. Calibration bar, 50 μm. ^*^P < 0.05, ^**^P < 0.01, ^***^P < 0.001.

Calretinin-expressing cells were distributed in the GrL/iPL and ML/ePL—where positive mitral cells have been included in the analysis—, but particularly in the GL/NL in both control and transgenic animals (Figures 5A–F). In the GrL/iPL of transgenic animals, the expression significantly increased from 30 (Figure 5A) to 56 (Figure 5B) weeks (Figure 5G) [Two-way ANOVA (genotype vs. age): Interaction *F*_(3, 28)_ = 3.334; *p* = 0.0336; Age *F*_(3, 28)_ = 1.875; *p* = 0.1568; Genotype *F*_(1, 28)_ = 1.481; *p* = 0.2338]. At 30 weeks, the expression in control animals was higher as compared to transgenic animals [*T*-test two tailed control vs. transgenic: t_7_ = 2.873; *p* = 0.0239]. In the ML/ePL of control animals, the expression significantly decreased from 16 (Figure 5C) to 30, 43, and 56 (Figure 5E) weeks (Figure 5G); and, at 16 weeks, the expression in control animals (Figure 5C) was higher as compared to transgenic (Figure 5D) animals (Figure 5F) [Two-way ANOVA (genotype vs. age): Interaction *F*_(3, 28)_ = 3.208; *p* = 0.0382; Age *F*_(3, 28)_ = 6.341; *p* = 0.0020; Genotype *F*_(1, 28)_ = 2.021; *p* = 0.1662] [*T*-test two tailed control vs. transgenic: *p* > 0.05]. In the GL/NL, no significant changes with aging or genotype were detected (Figures 5A–G) [Two-way ANOVA (genotype vs. age): Interaction *F*_(3, 28)_ = 1.031; *p* = 0.3940; Age *F*_(3, 28)_ = 0.7346; *p* = 0.5402; Genotype *F*_(1, 28)_ = 0.1791; *p* = 0.6754].

**Figure 6:**
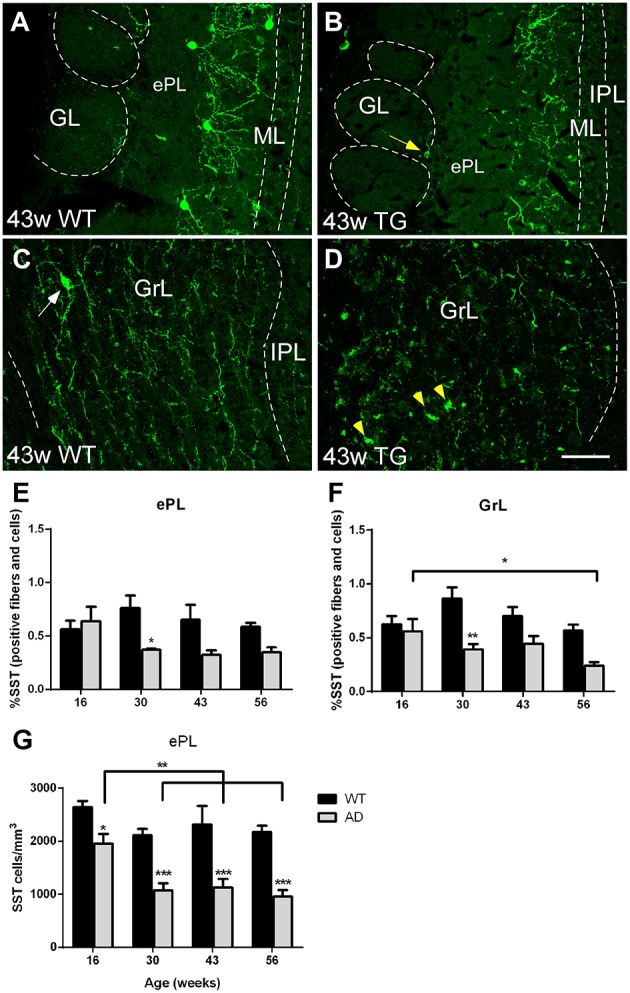
**Vulnerability analysis of somatostatin-expressing cells and fibers in the olfactory bulb. (A–D)**, Confocal images showing somatostatin-positive cells and fibers in the different layers of control and transgenic animals at distinct ages. **(E–G)**, graphics illustrating both % of area occupied by somatostatin cell bodies and fibers within ePL and GrL and the estimation of total somatostatin-positive in the ePL (cells/mm3) in control and transgenic animals. Calibration bar, 50 μm. White arrows point to labeled cells. Yellow arrow point to labeled periglomerular cell and yellow arrowheads to cell debries. ^*^P < 0.05, ^**^P < 0.01, ^***^P < 0.001.

Finally, regarding somatostatin-expression, cell bodies were mostly present in the ePL (Figure 6A) and only occasionally in the GrL (Figure 6C). Cell bodies degenerate with the disease leaving cell debris (Figure 6A vs. Figure 6B and Figure 6C vs. Figure 6D). To address, if cell bodies and/or fibers were reduced in different degree, we quantified the % of area within each region of interest occupied by somatostatin labeling (including cell bodies plus fibers) and total somatostatin positive cell bodies in the ePL. In this area, the reduction in % of area occupied by fibers and cell bodies starts early (at 30 weeks of age) and the reduction of somatostatin cell bodies is even sooner (at 16 weeks of age), suggesting that soluble pathology focuses on cell bodies (see discussion) (Figure 6E). Analysis of positive fibers and cells in the ePL [Two-way ANOVA (genotype vs. age): Interaction *F*_(3, 27)_ = 2.308; *p* = 0.0990; Age *F*_(3, 27)_ = 0.8809; *p* = 0.4633; Genotype *F*_(1, 27)_ = 10.61; *p* = 0.0030], GrL [Two-way ANOVA (genotype vs. age): Interaction *F*_(3, 27)_ = 2.027; *p* = 0.1338; Age *F*_(3, 27)_ = 2.864; *p* = 0.0552; Genotype *F*_(1, 27)_ = 22.68; *p* < 0.0001] and cell bodies in the ePL [Two-way ANOVA (genotype vs. age): Interaction *F*_(3, 27)_ = 0.9887; *p* = 0.4129; Age *F*_(3, 27)_ = 7.861; *p* = 0.0006; Genotype *F*_(1, 27)_ = 71.01; *p* < 0.0001] reveal significant lower expression in transgenic vs. control animals (Figure 6E). Further, in transgenic animals, a significant decrease with aging was observed in the expression of fibers and cells in the GrL [Two-way ANOVA (genotype vs. age): Interaction *F*_(3, 27)_ = 2.027; *p* = 0.1338; Age *F*_(3, 27)_ = 2.864; *p* = 0.0552; Genotype *F*_(1, 27)_ = 22.68; *p* < 0.0001] and in the expression of cell bodies in the ePL [Two-way ANOVA (genotype vs. age): Interaction *F*_(3, 27)_ = 0.9887; *p* = 0.4129; Age *F*_(3, 27)_ = 7.861; *p* = 0.0006; Genotype *F*_(1, 27)_ = 71.01; *p* < 0.0001] (Figures 6E–G).

### Analysis of Aβ

**Figure 7:**
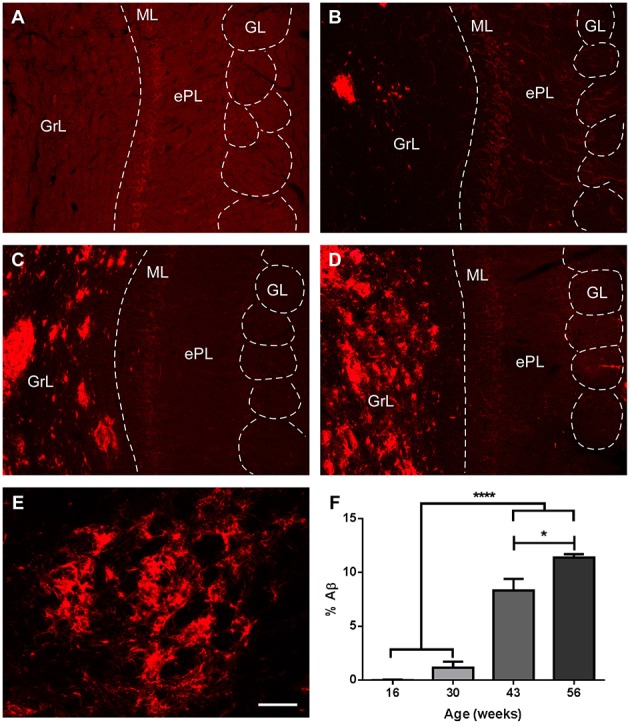
**Amyloid β aggregation over time in the olfactory bulb of transgenic animals. (A–D)**, %Aβ aggregates in transgenic mice at 16, 30, 43, and 56 weeks, respectively. **(E)**, high power of a plaque. **(F)**, graphics illustrating % of area occupied by Aβ, virtually restricted to the granule cell layer. Calibration bar for **(A–D)**, 80 μm, for E, 20 μm. ^*^P < 0.05, ^****^P < 0.0001.

As expected in the transgenic model, Aβ aggregation increased over time, but mostly restricted to the granule cell layer (Figures 7A–E). Percentage of area occupied by Aβ within GrL was significantly increased from 16 and 30 weeks vs. 43 and 56 weeks and from 43 to 56 weeks (Figure 7F) [One way ANOVA age: *F*_(3, 13)_ = 91.87, *p* < 0.0001].

### Co-localization of Aβ, interneuron markers, and BrdU

Since analysis of interneuron vulnerability revealed that calretinin-positive and, particularly, somatostatin-positive cells were decreased in transgenic animals, additional colocalization experiments were carried out to provide qualitative observations (see Supplementary Figure 2). Examples of somatostatin-positive cells with a dystrophic appearance (Supplementary Figure 2A) were placed in Aβ plaques (Supplementary Figure 2B) surrounded by Aβ aggregates (Supplementary Figure 2C). Calretinin-positive cells (Supplementary Figure 2D) were also located in Aβ plaques (Supplementary Figures 2E,F).

The low rate of BrdU-labeled cells coexpressing interneuron markers prevented to carry out quantitative analysis. Only a small percentage of them were observed co-expressing somatostatin or calretinin (Supplementary Figure 3) and amyloid β (see Supplementary Figure 4).

## Discussion

In the present report, neurogenesis, neurodegeneration, interneuron vulnerability, and amyloid β involvement has been investigated in the OB of mouse model of AD and control animals over time. The main results include: neurogenesis increases with aging in the granule cell layer of control animals from 16 to 43 weeks. No neurodegeneration changes have been observed after quantifying NeuN or PGP9.5. Calbindin-expressing neurons remains unchanged. Parvalbumin-expressing neurons trend to increase with aging in transgenic animals. Calretinin-expressing neurons increase with aging in transgenic mice in the GrL and decrease in control animals in the ML and it is higher in control as compared to transgenic animals at given ages. Somatostatin-expressing neurons of transgenic mice decrease with aging and abruptly as compared to controls. Amyloid β aggregates with aging in the granule cell layer, which may be related to the particular involvement of somatostatin-expressing cells.

Therefore, neurogenesis in transgenic animals, in contrast to control animals, do not increase with aging. Regarding interneuron vulnerability, calbindin expression is not altered, parvalbumin expression is increased, calretinin increases in transgenic and decreases in control animals and somatostatin is strongly reduced with aging and in transgenic animals as compared to controls. This evidences differential vulnerability among interneuron pupulations which may be related to Aβ pathology.

### Neurogenesis in Alzheimer's disease

Fifty years after the first seminal report of neurogenesis in the adult brain (Altman and Das, 1965), the specific role of new-born cells integrated in the DG (Kempermann et al., 2015) or OB (Lepousez et al., 2015) is not fully understood. Growing interest has focused on therapeutic opportunities, particularly regarding neurodegenerative diseases, in particular Huntington's, Parkinson's, and Alzheimer's diseases (Kaneko and Sawamoto, 2009; Lazarov et al., 2010; Winner et al., 2011; Winner and Winkler, 2015).

In the case of AD, there is a number of molecules implicated in its etiology that also play a key role in adult neurogenesis such as ApoE, PS1, APP, and its metabolites (Lazarov and Marr, 2010; Mu and Gage, 2011). In particular, it has been demonstrated that soluble APPα regulates neural progenitor cell proliferation and that miscleavage of APP could greatly influence in developmental and postnatal neurogenesis, which could contribute to symptomatic cognitive deficits in AD (Lazarov and Demars, 2012).

Data on neurogenic rates are quite variable depending on species, age, environment, and disease. Neurogenesis occurs in postnatal and adult rodents and postnatal humans (Lazarov and Marr, 2013) but, in adult humans, there is substantial hippocampal and striatal neurogenesis and it is no detectable in the OB (Bergmann et al., 2015). There is a general agreement regarding reduction of neurogenesis with aging (Hamilton et al., 2013), which contrast with our present results in control animals. In the granule cell layer, it has been described a decreasing number of new-born cell with aging (Petrenau and Alvarez-Buylla, 2002; Winner et al., 2002), which does not match our results. It is interesting to note that previous reports show non-significant peaks at 20 (Petrenau and Alvarez-Buylla, 2002) and 24 weeks (Winner et al., 2002) and ours results a significant peak at 43 weeks. Likely these discrepancies are due methodological differences regarding the method used: BrdU vs. [^3^H]-thymidine or BrdU administration in 1 day vs. 4 consecutive days. In diseased brains, however, data are highly variable depending on the pathology analyzed. Even in AD, data are controversial depending on model used, experimental conditions, markers or area studied (Lazarov and Marr, 2010; Winner et al., 2011).

Reports in the double APP/PS1 transgenic model show a reduced hippocampal neurogenesis. Niidome and colleagues describe no reduction of proliferating cells in the SVZ, but a significant decrease in the SGZ (Niidome et al., 2008). Verret and colleagues report that, although hippocampal proliferation was unaffected, survival of newborn cells 4 weeks later was dramatically diminished (Verret et al., 2007). Conversely, Demars and colleagues, conclude that this neurogenesis impairment occurs early in life long before amyloid deposition (Demars et al., 2010). Interestingly, it has been reported that the reduction in neuroblasts was restricted to the hippocampal but not to the OB neurogenic system (Zhang et al., 2007). In fact, our results (Figure 1) show no significant reduction in the number of BrdU-labeled cells in the glomerular layer in APP/PS1 model.

### Amyloid β proteinopathy and interneuron population involvement

Alzheimer disease is a neurodegenerative disease and as such, neuronal loss is expected. It is difficult, however, to distinguish neurodegenerative changes that accompany normal aging (Attems et al., 2015) from those that characterize AD. In the hippocampus, for instance, a significant cell loss was reported in CA1 in Alzheimer's patients, whereas there was almost no neuron loss in the normal aging group (West et al., 1994). In the OB, it has been reported that the total number of cells and the number of mitral cells were the same for controls and patients, but the volume of the bulb and the number of cells in the anterior olfactory nucleus was reduced up to 75% in younger patients (Ter Laak et al., 1994). Our results on the number of NeuN- and PGP9.5-positive cells in the different layers of the bulb reveal no neuronal loss with aging in both control and transgenic mice suggesting that volume reduction must be due to neuropil reorganization and shrinkage.

Amyloid β aggregation in the OB has been described as an early event in the neuropathology of AD (Ohm and Braak, 1987; Jellinger and Attems, 2005; Attems et al., 2012, 2014). In APP/PS1 mouse model, Aβ aggregation has been reported in the olfactory cortex (Saiz-Sanchez et al., 2012) as well as in the OB and anterior olfactory nucleus (Saiz-Sanchez et al., 2013). The present data are in agreement with these reports showing an accumulative aggregation of Aβ plaques in the OB, but restricted to the granule cell layer.

It has been described that the different interneuron populations show differential expression and vulnerability in the disease as compared to controls in given areas (Iritani et al., 2001; Saiz-Sanchez et al., 2016) and not in others (Leuba et al., 1998). In human tissue, it has been reported a 50% reduction in somatostatin expression in the anterior olfactory nucleus matching a high co-localization with Aβ (Saiz-Sanchez et al., 2010). In the piriform cortex, somatostatin and calretinin expression was reduced showing high co-localization with Aβ, whereas parvalbumin expression was increased (Saiz-Sanchez et al., 2015) in agreement with our present results. It is interesting to note that not only Aβ aggregates, but soluble Aβ—not detected with the used antibody—is particularly toxic for neurons. Other authors describes that calretinin-positive cells are resistant to neurodegeneration in the human temporal cortex (Fonseca and Soriano, 1995)—which matches the light increase described herein—and other authors reports differential vulnerability of parvalbumin-positive cells depending on the hippocampal field (Brady and Mufson, 1997) or entorhinal cortex (Solodkin et al., 1996; Mikkonen et al., 1999). In the olfactory cortex of APP/PS1 model, similar results were found being somatostatin and calretinin expression severely reduced and calbindin and parvalbumin expression later and only moderately reduced (Saiz-Sanchez et al., 2012). It is interesting to note that previous reports in our laboratory (Saiz-Sanchez et al., 2012, 2013) studied groups up to 8 months of age, whereas in the present report these observations were extended up to 56 weeks. Therefore, present data are in agreement with previous reports and enlarge previous descriptions reveling, for instance, a late increase of parvalbumin expression that could constitute a compensatory mechanism of some calcium binding protein expressing interneuron populations. In the OB, previous results were similar (Saiz-Sanchez et al., 2013) and in agreement with present data were calretinin- and, particularly somatostatin-, expression was reduced in transgenic as compared to control animals.

Regarding Alzheimer's etiology, it has been described that decreased somatostatin expression may predispose to Aβ accumulation. This finding has raised the possibility that somatostatin receptor agonists may be of therapeutic value in AD (Hama and Saido, 2005; Iwata et al., 2005; Saito et al., 2005; Saido and Iwata, 2006). The present results further support the idea that somatostatin expressing neurons are early and preferentially involved by Aβ pathology in AD and that this could be on its etiology.

## Author contributions

CD experiments and analysis. DS, IU, AF analysis and quantification. AM coordination and writing.

### Conflict of interest statement

The authors declare that the research was conducted in the absence of any commercial or financial relationships that could be construed as a potential conflict of interest.

## Abbreviations

Aβ: amyloid β
AD: Alzheimer's disease
APP: amyloid precursor protein
BrdU: bromodeoxyuridine
CB: calbindin
CR: calretinin
DG: dentate gyrus
ePL: external plexiform layer
GL: glomerular layer
GrL: granule cell layer
iPL: internal plexiform layer
MOB: main olfactory bulb
NeuN: Fox3-NeuN
OB: olfactory bulb
PS1: presenilin 1
PV: parvalbumin
SGZ: subgranular zone
SST: somatostatin
SVZ: subventricular zone
TG: transgenic
WT: wild type.
