# Supplementary material for: Neurogenesis, Neurodegeneration, Interneuron Vulnerability, and Amyloid-β in the Olfactory Bulb of APP/PS1 Mouse Model of Alzheimer's Disease
Source: Front Neurosci. 2016 May 30;10:227. doi: 10.3389/fnins.2016.00227 (PMC4885141; doi:10.3389/fnins.2016.00227)
Supplement: Supplementary file 1 [file Image1.PDF]

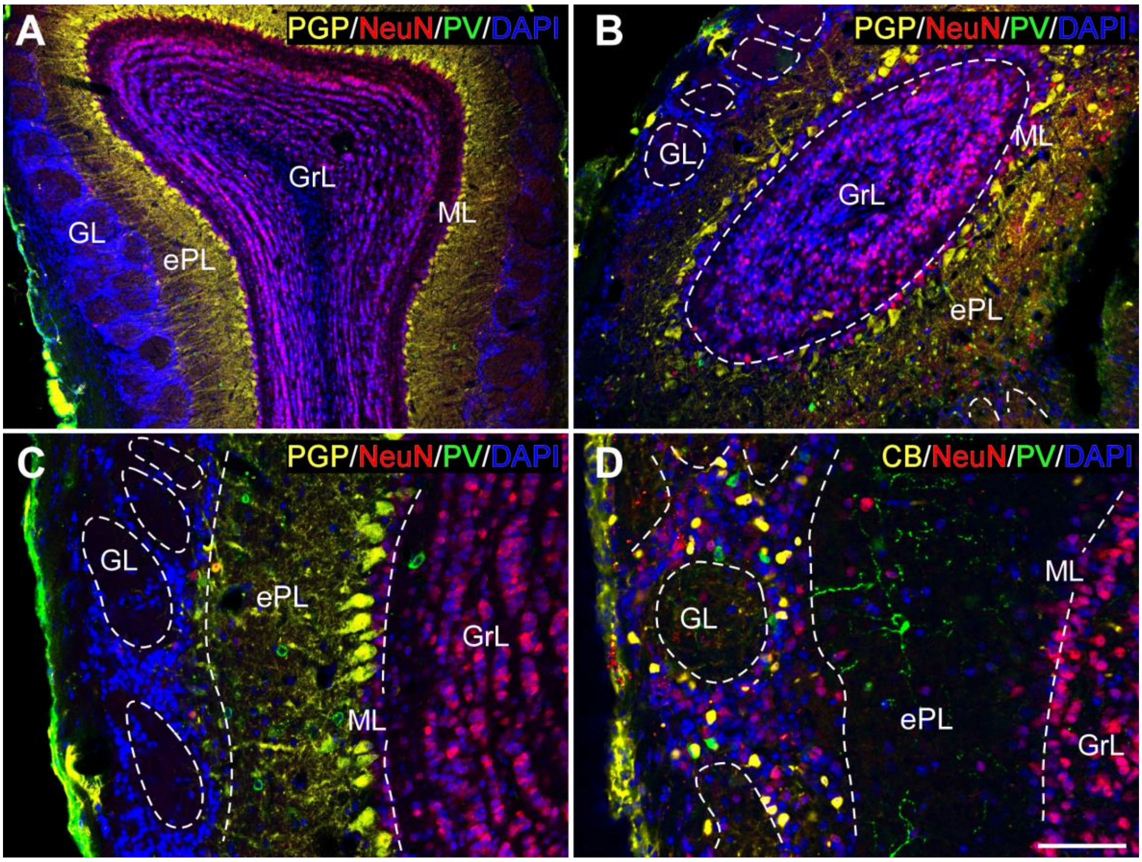

Supplementary figure 1.- A-C, Quadruple staining against PGP9.5, NeuN, parvalbumin and DAPI at low magnification (A) and high magnification (B and C). D, quadruple staining against calbindin, NeuN, parvalbumin and DAPI at high magnification. Scale bar: A, 200um; B, 100um; C and D, 50um.

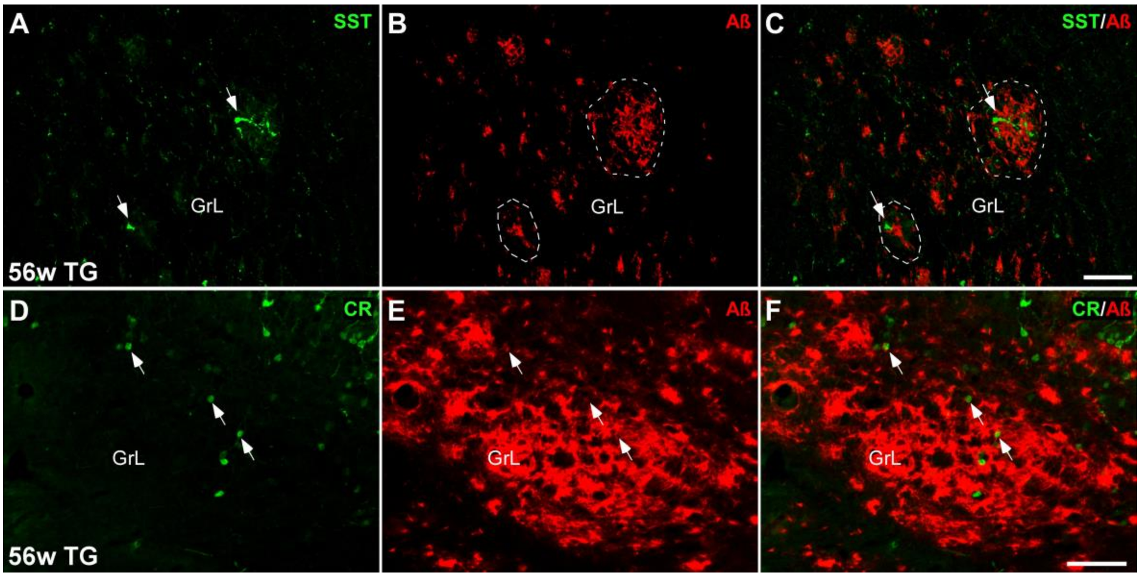

Supplementary figure 2.- A-C, confocal images showing resulting somatostatin labeling and colocalization with amiloid β in the granule cell layer of transgenic mice at 56 weeks. D-F, confocal images illustrating calretinin-positive cells and colocalization with amiloid β in granule cell layer of transgenic mice at 56 weeks. Calibration bar, 50 µm.

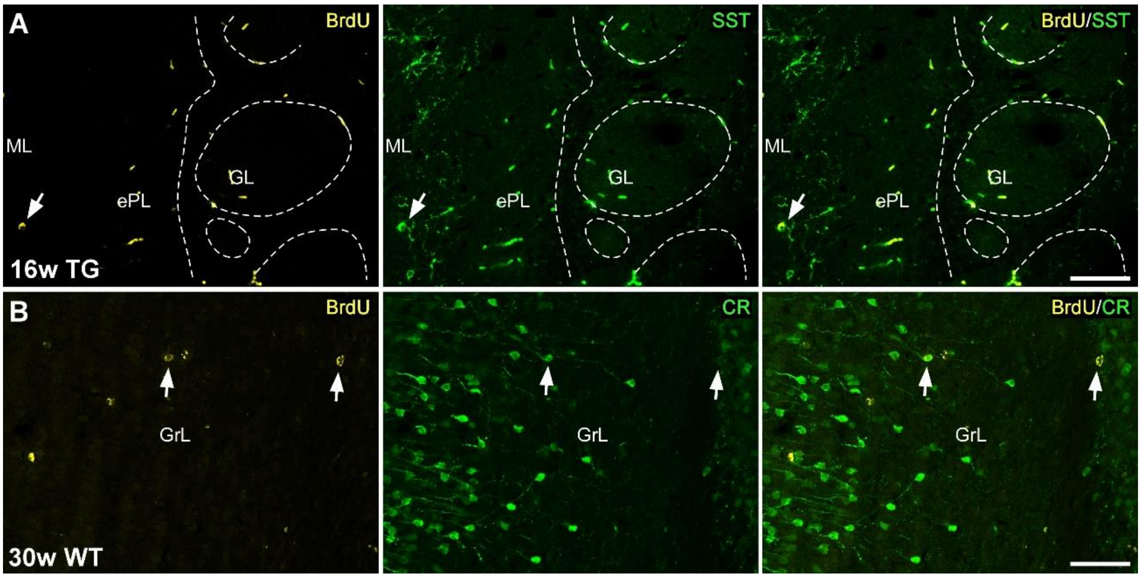

Supplementary figure 3.- A, shows bromodeoxyuridine-positive cell also positive for somatostatin marker (arrow) in the mitral/external plexiform cell layer of transgenic mice at 16 weeks. B, shows multiple bromodeoxyuridine-positive cells also positive for calretinin (arrows) in the granular cell layer of control animals at 30 weeks. Calibration bar, 50 µm.

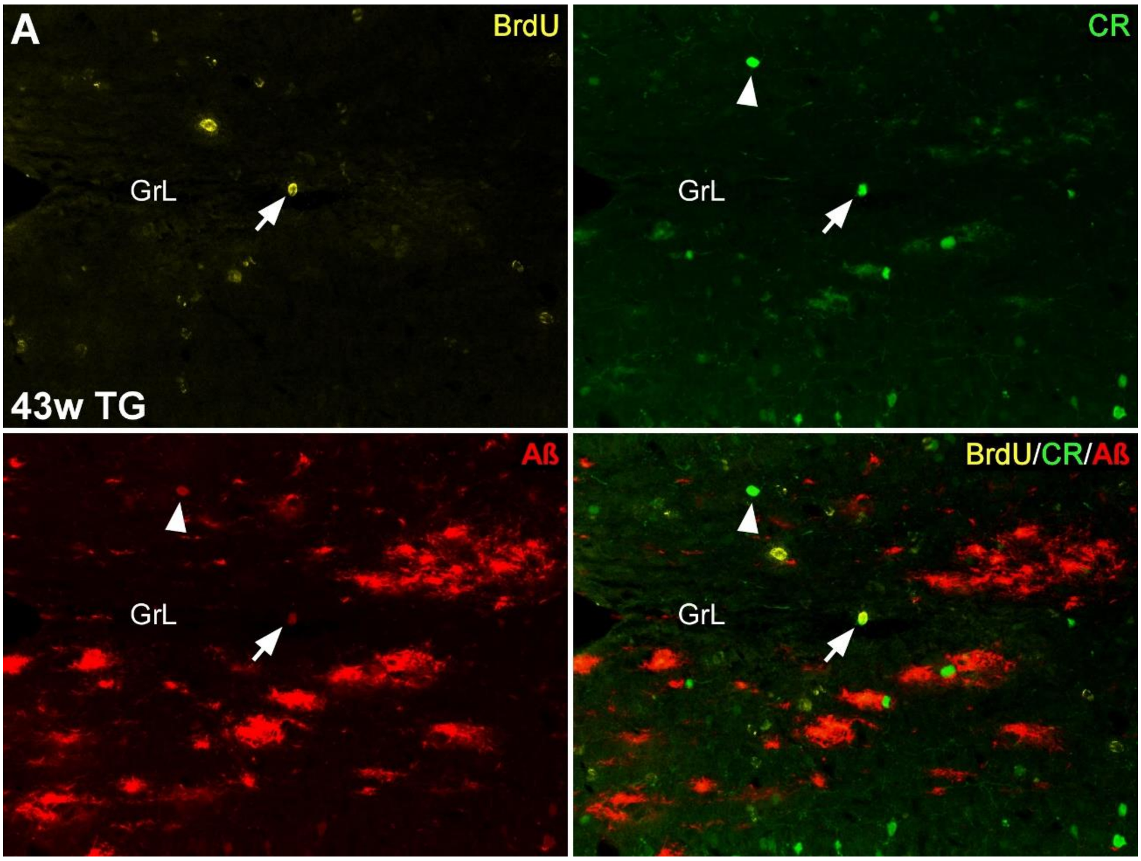

Supplementary figure 4.- A, confocal images showing bromodeoxyuridin-positive cells (arrows) and calretinin-positive cells (arrowheads) colocalizing with amiloid β in the granule cell layer of transgenic animals at 43 weeks. Calibration bar, 50 µm.
